# Fabrication of a P3HT-ZnO Nanowires Gas Sensor Detecting Ammonia Gas

**DOI:** 10.3390/s18010037

**Published:** 2017-12-25

**Authors:** Chin-Guo Kuo, Jung-Hsuan Chen, Yi-Chieh Chao, Po-Lin Chen

**Affiliations:** Department of Industrial Education, National Taiwan Normal University, 162, Sec.1, Heping E. Rd., Taipei 10610, Taiwan; chinguo7@yahoo.com.tw (C.-G.K.); ycchao@ntnu.edu.tw (Y.-C.C.); 60670024h@ntnu.edu.tw (P.-L.C.)

**Keywords:** poly(3-hexylthiophene), zinc oxide nanowire, gas sensor, ammonia gas

## Abstract

In this study, an organic-inorganic semiconductor gas sensor was fabricated to detect ammonia gas. An inorganic semiconductor was a zinc oxide (ZnO) nanowire array produced by atomic layer deposition (ALD) while an organic material was a p-type semiconductor, poly(3-hexylthiophene) (P3HT). P3HT was suitable for the gas sensing application due to its high hole mobility, good stability, and good electrical conductivity. In this work, P3HT was coated on the zinc oxide nanowires by the spin coating to form an organic-inorganic heterogeneous interface of the gas sensor for detecting ammonia gas. The thicknesses of the P3HT were around 462 nm, 397 nm, and 277 nm when the speeds of the spin coating were 4000 rpm, 5000 rpm, and 6000 rpm, respectively. The electrical properties and sensing characteristics of the gas sensing device at room temperature were evaluated by Hall effect measurement and the sensitivity of detecting ammonia gas. The results of Hall effect measurement for the P3HT-ZnO nanowires semiconductor with 462 nm P3HT film showed that the carrier concentration and the mobility were 2.7 × 10^19^ cm^−3^ and 24.7 cm^2^∙V^−1^∙s^−1^ respectively. The gas sensing device prepared by the P3HT-ZnO nanowires semiconductor had better sensitivity than the device composed of the ZnO film and P3HT film. Additionally, this gas sensing device could reach a maximum sensitivity around 11.58 per ppm.

## 1. Introduction

In past decades, gas sensors based on metal oxide semiconductors have drawn much attention for wide applications [1,2,3,4,5] such as environmental monitoring, industrial process control, and for the detection of the chemical or toxic substrates. Recently, the gas sensors composed of nanostructured semiconductors, such as nanowires, nanoparticles, nanorods, and nanobelts, show better sensitivities to different gases [6,7,8,9]. The sensors with nanostructures have been demonstrated to be excellent candidates for high sensitivity due to their high surface to volume ratio. However, some limitations of these sensors based on metal oxide semiconductors, like high working temperature could be seen [10]. 

Organic semiconductors have potential to be used as the sensing layer of the gas sensor because they have good gas sensing performance and can be used to fabricate the low cost gas sensors. High selectivity for gas and operating in the lower temperature are the most notable advantages for the organic gas sensors [11,12]. Poly(3-hexylthio-phene) (P3HT) is a widely investigated conductive conjugated polymer and exhibits a tendency to self-assemble into polycrystalline structure when casts from the solution phase [13]. P3HT is a very stable p-type semiconductor with holes as carriers and usually used in the organic thin film transistor (OTFT) sensors. A. Assadi and his coworkers [14] proposed a P3HT based field effect transistor (FET) in 1990 and found that changes in electrical properties of FET upon exposure to alumina gas were reversible. Fukuda et al. [15] showed that the regioregular P3HT thin film transistor was sensitive to 1000 ppm of N_2_O gas exposure at 50 °C. Jeong et al. [16] reported that response characteristics of P3HT based OTFT sensors exposed to ammonia gas with concentrations ranging from 10 to 100 ppm at room temperature in normal atmosphere. Additionally, Saxena et al. [10] demonstrated that P3HT: ZnO nanowire hybrid films exhibited high sensivity, fast response, and enhanced selectivity for NO_2_ gas in the 0–10 ppm range at room temperature. Organic-inorganic hybrid materials exhibited good mechanical and electrical properties, and could be used in a selective gas sensor.

In the present study, an ammonia gas sensor consisting of the P3HT film and the ZnO nanowires was fabricated. P3HT film was coated on the surface of the ZnO nanowires by spin coating technique and then an organic-inorganic heterojunction was formed. Combining the benefits of two materials, it is believed that this structure would exhibit higher electrical properties and more sensitive performance for gas sensing. The findings of this study will provide helpful information for designing and fabricating highly sensitive gas sensors with organic-inorganic semiconductors.

## 2. Materials and Methods

**Figure 1:**
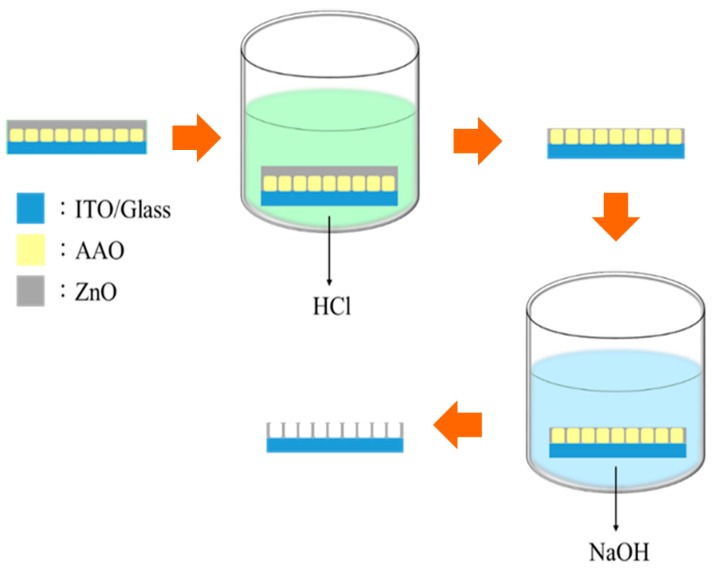
The process flow of removing the anodic aluminum oxide (AAO) template by the wet etching method.

An aluminum film was coated on a conductive glass of indium tin oxide (ITO) using RF magnetron sputter. Then a two-step anodization treatment was adopted to prepare the anodic aluminum oxide (AAO) template. First step of the anodization was carrying out in 0.3 M oxalic acid solution by applying 40 volts at 0 °C for 30 s. The as-prepared oxide layer was then removed using a mixture solution of 3% chromic acid solution (CrO_3_) and 6% phosphoric acid solution (H_3_PO_4_) at 60 °C. Second step of the anodization was performed under the same condition as the first anodization, but anodizing time became 1.5 min. Finally, a pore widening process was executed using 5% phosphoric acid solution. In this study, the ZnO nanowires were fabricated within the AAO template by the atomic layer deposition (ALD). The AAO/ITO specimen rinsing with the deionized (DI) water and acetone solution was placed in a chamber under a high vacuum of 10^−6^ Torr. ZnO was then deposited into the nanopores of the AAO template. It was difficult to know when the nanopore was filled with ZnO, therefore the excess ZnO would become a ZnO film on the surface of the AAO template after the ALD process. In order to remove the extra ZnO film, a wet etching was performed using the hydrochloric acid (HCl) solution. After that, the AAO template dissolved in the sodium hydroxide (NaOH) solution and the ZnO nanowires with a high aspect ratio were exposed, as shown in Figure 1. For details of the procedure for the formation of the ZnO nanowires, refer to our previous paper [17].

Spin coating technique was adopted to prepare a uniform P3HT film on the surface of the ZnO nanowires. P3HT was dissolved in an organic solvent, tetrahydrofuran (THF), and produced a 1wt.% P3HT solution for spin coating. In order to obtain a smooth and well distributed P3HT film on the surface of the nanowires, the spin coating process was divided into two stages. At the first stage, the rotation speed was 500 rpm (round per minute) for 5 s. Then, the rotation speed was raised to various speeds of 4000, 5000, and 6000 rpm for 30 s to prepare a uniform P3HT film on the surface of the ZnO nanowires.

**Figure 2:**
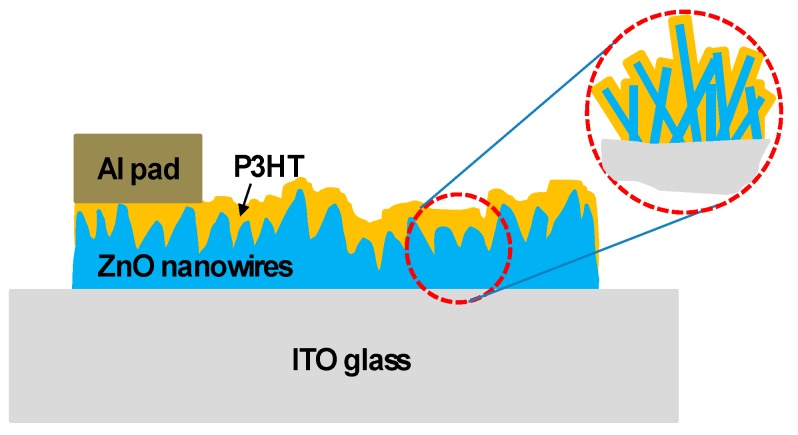
Schematic diagram of the sensing device.

**Figure 3:**
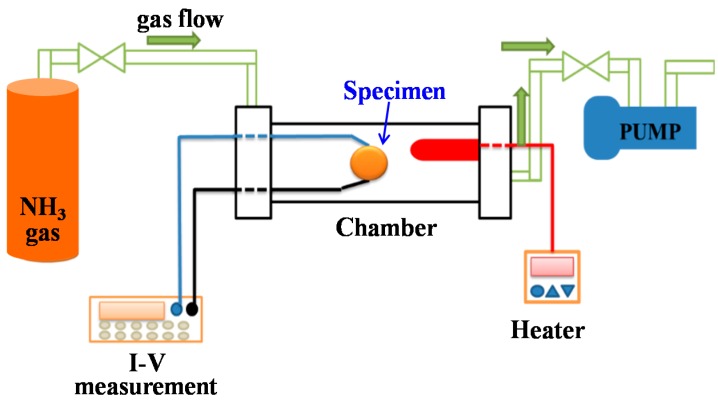
The equipment for gas sensing.

The P3HT-ZnO nanowires (organic-inorganic semiconductors) gas sensor could be obtained after aluminum electrodes deposited on the surface of the P3HT film by the thermal evaporator and the schematic diagram of the sensing device was shown in Figure 2.The sensing properties of the prepared P3HT-ZnO nanowires gas sensor were measured at room temperature under various concentrations of ammonia gas and the gas detection system was illustrated in Figure 3. In this study, the morphologies of the AAO template and the ZnO nanowires were inspected by scanning electron microscopy (SEM) and crystalline structure of the ZnO nanowires was carried out using X-ray diffraction (XRD) analysis. The carrier concentration and the mobility of the semiconductor composed of the P3HT and the ZnO nanowires were determined by Hall effect measurement.

## 3. Results and Discussion

**Figure 4:**
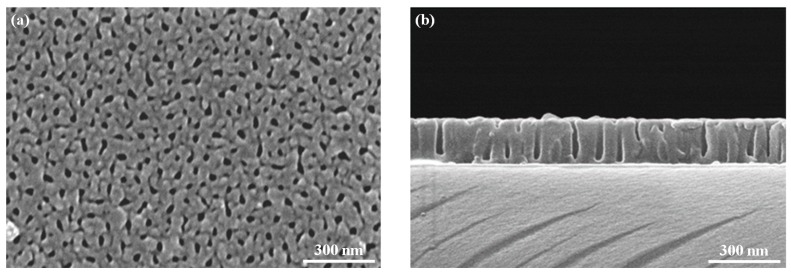
Scanning electron microscope (SEM) images of the AAO template: (**a**) top view; (**b**) cross-sectional view.

**Figure 5:**
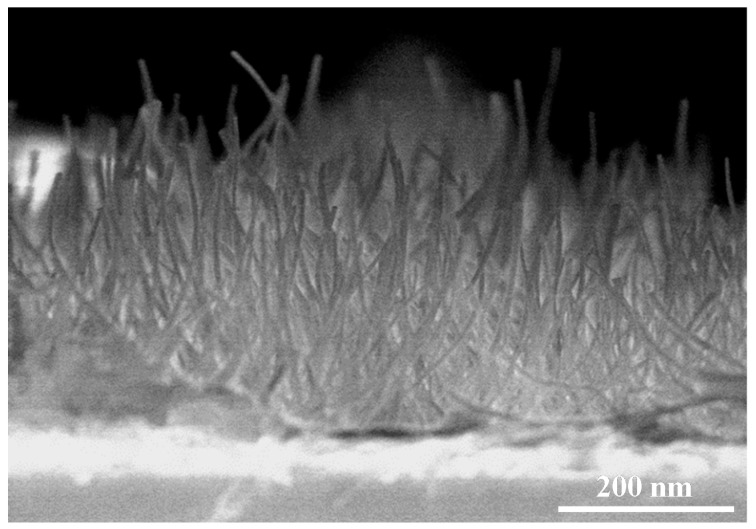
Zinc oxide (ZnO) nanowires without the AAO template which was removing by immersing in 0.1 M sodium hydroxide (NaOH) solution for 10 min.

**Figure 6:**
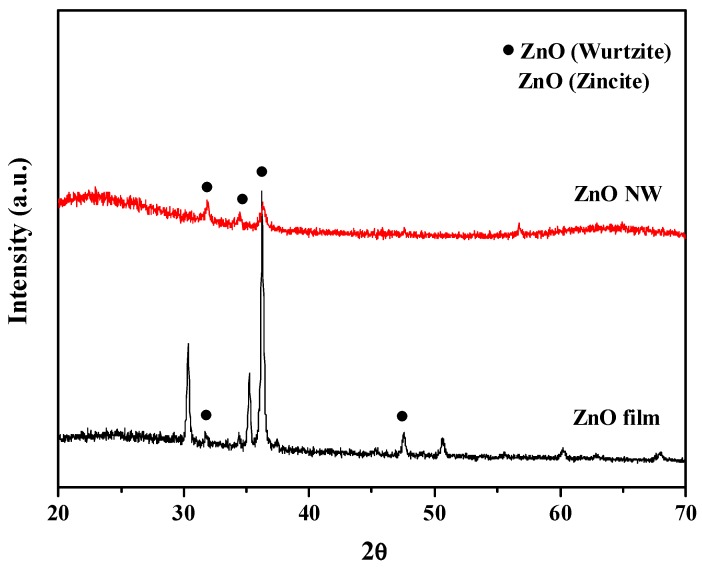
X-ray diffraction (XRD) results of the ZnO film and the ZnO nanowires.

The AAO template is a self-assembled nanoporous film in which the pore size and the distribution are influenced by the working voltage, electrolyte compositions, and other parameters [18,19,20,21]. In the present work, the AAO template was produced on the ITO glass in 0.3 M oxalic acid solution at 40 V. Figure 4a showed the surface morphology of the AAO template after a pore widening process using 5% phosphoric acid solution. The straight nanopore was found through the cross-sectional view of the AAO template as showed in Figure 4b. ZnO was deposited into the nanopores of the AAO template by the ALD process. The ZnO nanowires could be obtained after the extra ZnO film which was capped the surface of the AAO template and the AAO template were removed using 0.1 M HCl solution for 10 s and 0.1 M NaOH solution for 10 min, respectively. Figure 5 revealed that the dense ZnO nanowires with a high aspect ratio were produced. The crystal structures of the ZnO nanowires and the ZnO film, which were both fabricated with the ALD technique, were examined by XRD analysis. The results in Figure 6 indicated that the ZnO film consisted of the wurtzite structure and the zincite structure. The spectrum of the ZnO nanowires showed the small diffraction signals of the wurtzite structure and a board amorphous signal might come from the residues of the AAO template [22].The small diffraction signals of wurtzite structure were contributed by the finer ZnO nanostructures and the confinement of the AAO template.

In order to fabricate an ammonia gas sensor device, the sensing layer, P3HT, was coated on the as-prepared ZnO nanowires by the spin coating method. The thickness of the P3HT could be adjusted by varying the rotation speed. In this work, various rotation speeds, 4000 rpm, 5000 rpm, and 6000 rpm were adopted and the corresponding thickness of P3HT film were respectively around 462 nm, 397 nm, and 277 nm. The results of Hall effect measurement for P3HT-ZnO nanowires semiconductor with 462 nm P3HT film showed that the carrier concentration and the mobility were 2.7 ×10^19^ cm^−3^ and 24.7 cm^2^∙V^−1^∙s^−1^. In comparison with the pure P3HT film which mobility was around 10^−3^ to 10^−2^ cm^2^∙V^−1^∙s^−1^, the significant increases of both carrier concentration and mobility of P3HT-ZnO nanowires semiconductor might be contributed from a synergetic effect of the organic and inorganic moieties. The similar phenomenon observed in P3HT-SnO_2_ composite semiconductor was reported by Geng and his coworkers [23].

The ammonia sensing properties of the device were measured by the resistance change under an ammonia gas ambient at various concentrations of 5 ppm, 1 ppm, 0.5 ppm and 0.1 ppm in this experiment. The gas response of the sensing device is defined by the ratio of the variation of the resistance change and the initial resistance, as showed in following formula.

(1) $$Response=\frac{\Delta R}{R_{0}}\times 100\%=\frac{R_{gas}-R_{0}}{R_{0}}\times 100\%$$

*R*_0_ is the initial resistance. *R_gas_* is the resistance of the gas sensor under the target gas ambient. Sensitivity (*S*) of the sensing device is defined as [24]:

(2) $$S=\frac{R_{\mathrm{gas}}/R_{0}}{C_{g}}$$

where *C_g_* refers to the gas concentration of the target gas. 

**Figure 7:**
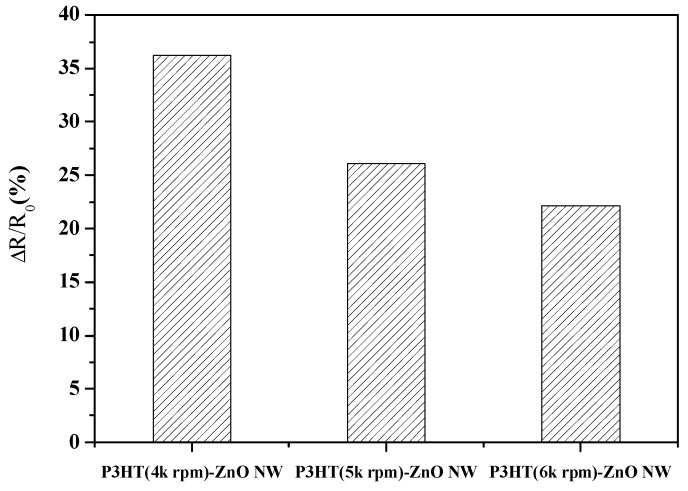
The responses of the poly(3-hexylthio-phene) (P3HT)-ZnO nanowires sensors with various thicknesses of the P3HT detecting 5 ppm ammonia gas.

**Figure 8:**
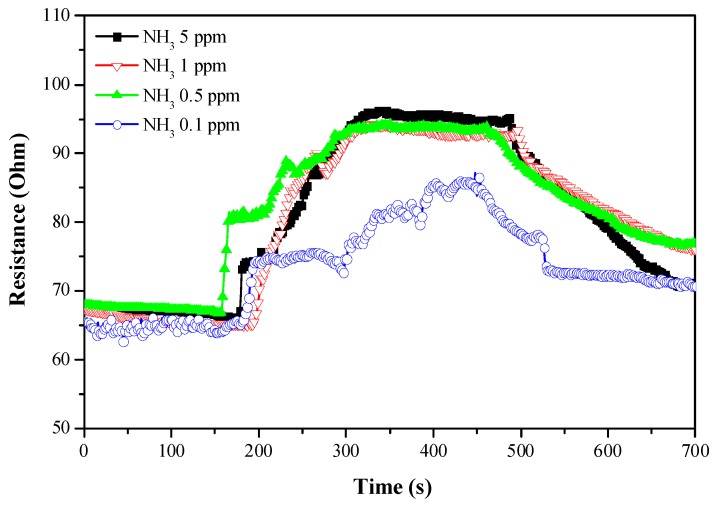
Dependence of resistance on time for the P3HT-ZnO naowires devices which the P3HT films were produced by the spin coating in 4000 rpm.

**Figure 9:**
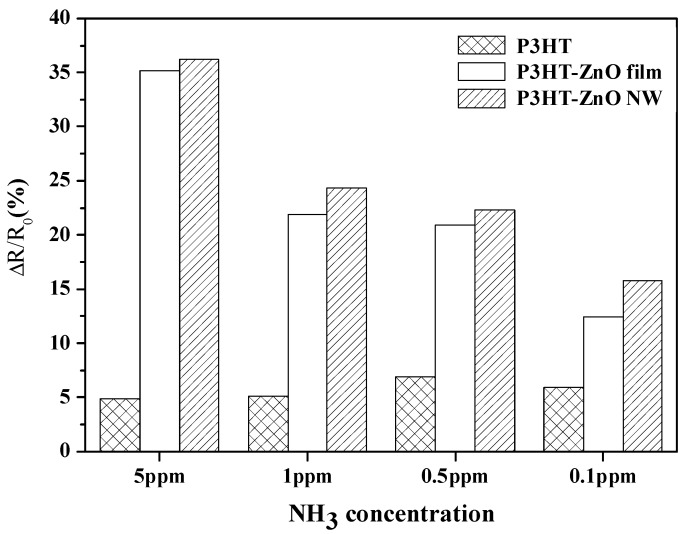
The comparisons of the responses of P3HT, P3HT-ZnO film and P3HT-ZnO nanowires when all P3HT films were produced by the spin coating in 4000 rpm.

Figure 7 showed the response of P3HT-ZnO nanowires sensors with various P3HT thicknesses to the 5 ppm ammonia gas. All three sensors showed responses to ammonia gas and the gas response of the P3HT-ZnO nanowires increased with increasing thickness of the P3HT.The sensor with 462 nm P3HT film produced by the rotation speed of 4000 rpm had the best gas response to ammonia gas among the three sensors. It was supposed that the thinner P3HT could not cover the nanowires well because the length of nanowires of several hundred nanometers was observed in the SEM image. The poor surface coverage would lead to poor gas sensing performance. Figure 8 displayed the dynamic responses of the P3HT-ZnO nanowires sensor with 462 nm P3HT film exposing to different concentrations of ammonia gases from 0.1 ppm to 5 ppm. Figure 9 showed the response of the P3HT-ZnO nanowires sensor with 462 nm P3HT film for different concentrations of ammonia gas. For comparison, the responses of the P3HT sensor and the P3HT-ZnO film sensor were also measured. The P3HT-ZnO nanowires sensor had the higher response to the ammonia gas in all the examined concentrations than the P3HT sensor and the P3HT-ZnO film sensor, and the maximum sensitivity was around 11.58 per ppm. Table 1 showed that the comparison of the sensitivity between the P3HT-ZnO nanowires sensor and previously reported ammonia gas sensors. The data indicated that the device fabricated in this work had the lowest detection limit and the best sensitivity among the organic sensing devices.

The P3HT-ZnO nanowires sensor could detect the low concentration of ammonia gas and the detection limit was lower than 0.1 ppm. Lower detection limit and high sensitivity were important for the ammonia gas sensor, especially for the diagnosis of liver cirrhosis [25,26]. Shimamoto and his coworkers [25] reported that breath ammonia concentrations were higher in cirrhotic patients (~0.75 ppm) than in healthy persons (~0.28 ppm). They also found that the breath ammonia concentration could correlate positively with the blood ammonia concentration. Therefore, the ammonia gas sensor composited of the P3HT and the ZnO nanowires fabricated in this study had the opportunity to be developed as a suitable device for the non-invasive diagnosis of liver diseases.

## 4. Conclusions

In this study, a gas sensor composed of the P3HT and the ZnO nanowires was successfully fabricated to detect ammonia gas. P3HT was coated onto the surface of the ZnO nanowires, which were produced by the ALD technique, using the spin coating process. The surface morphology and crystal structure of the ZnO nanowires produced in this work were analyzed by SEM and XRD. Experimental results showed that the ZnO nanowire with the wurtzite crystal structure was obtained and did not get damaged after the wet etching process. After the P3HT film was coating the surface of the ZnO nanowires, the Hall effect measurement revealed that the carrier concentration and the mobility were 2.7 ×10^19^ cm^−3^ and 24.7 cm^2^∙V^−1^∙s^−1^ respectively. There were significant increases in both carrier concentration and mobility of the P3HT-ZnO nanowires semiconductor, and these might be caused by the synergetic effect of the organic and inorganic moieties. Gas sensing properties at room temperature showed that the device produced by the P3HT-ZnO nanowires in which the P3HT film was 462 nm in thickness had the maximum sensitivity of 11.58 per ppm, and its detection limit was lower than 0.1 ppm.

## Figures and Tables

**Table 1:** Sensing properties of the presented sensor in this work compared with other ammonia gas sensors (previous works).

Sensing Materials	Working Temperature	Concentration (ppm)	Maximum Sensitivity ** (per ppm)	Ref.
rGO*-ZnO bilayer thin film	RT	50	0.0206	[27]
MoS_2_-ZnO nanocomposite	RT	0.25~100	0.0292	[28]
PPy coated TiO2-ZnO nanofiber	RT	0.5~450	0.2323	[29]
Fe_2_O_3_-ZnO nanocomposite	RT	0.4	25,000	[30]
PANI-ZnO hybrid film	RT	10~50	0.0302	[24]
SnO_2_-ZnO-PPy multilayer	RT	30~70	0.0239	[31]
P3HT-ZnO nanowires	RT	0.1~5	11.5762	This work

* rGO: reduced graphene oxide; ** Sensitivity: (*R_gas_*/*R*_0_)/gas concentration.
